# Increased ratio of sST2/LVMI predicted cardiovascular mortality and heart failure rehospitalization in heart failure with reduced ejection fraction patients: a prospective cohort study

**DOI:** 10.1186/s12872-021-02191-3

**Published:** 2021-08-17

**Authors:** Fuhai Li, Mengying Xu, Mingqiang Fu, Xiaotong Cui, Zhexun Lian, Hui Xin, Jingmin Zhou, Junbo Ge

**Affiliations:** 1 Department of Cardiology, The Affiliated Hospital of Qingdao University, Qingdao, 266003 China; 2 Department of Cardiology, Shanghai Institute of Cardiovascular Diseases, Zhongshan Hospital, Fudan University, Shanghai, 200032 China

**Keywords:** Soluble suppression of tumorigenicity 2, Left ventricular mass index, Heart failure, Cardiac remodeling, Heart failure with reduced ejection fraction

## Abstract

**Background:**

Inflammation is one of the principal triggering mechanisms for left ventricular fibrosis and remodeling in heart failure, leading to adverse clinical outcomes. Soluble suppression of tumorigenicity 2 (sST2), a member of the interleukin-1 receptor family, is assumed to play a significant role in the fibrotic response to inflammation. Left ventricular mass index (LVMI) is a parameter of the prefibrotic inflammatory phase of heart failure preceding remodeling. The present study aimed to investigate the prognostic value of the sST2/LVMI ratio in heart failure with reduced ejection fraction.

**Methods:**

This was a prospective cohort study. A total of 45 consecutive patients with heart failure with reduced ejection fraction, treated between September 2015 and December 2016, were enrolled. The sST2/LVMI ratio was measured at baseline. The primary endpoint was a composite of cardiovascular mortality and readmission for heart failure. The prognostic impact of the sST2/LVMI ratio was evaluated using a multivariable Cox proportional hazards regression model.

**Results:**

Forty-five patients were enrolled in this study. Their average age was 48 ± 14 years, and approximately 20% of them were men. Patients were followed for 9 months, during which the primary outcome occurred in 15 patients. Kaplan–Meier analysis showed that patients with a high sST2/LVMI ratio (≥ 0.39) had shorter event-free survival than those with intermediate (between 0.39 and 0.24) and low ratios (< 0.24) (log-rank, *P* = 0.022). The fully adjusted multivariable Cox regression analysis showed that the sST2/LVMI ratio was positively associated with the composite outcome in patients with heart failure with reduced ejection fraction after adjusting for confounders (hazard ratio 1.64, 95% confidence interval 1.06 to 2.54). By subgroup analysis, a stronger association was found with age between 40 and 55 years, systolic blood pressure < 115 or ≥ 129 mmHg, diastolic blood pressure < 74 mmHg, hematocrit < 44.5%, and interventricular septum thickness ≥ 8.5 mm.

**Conclusion:**

In patients with heart failure with reduced ejection fraction, the relationship between the sST2/LVMI ratio and the composite outcome was linear. A higher baseline ratio of sST2/LVMI was associated with an increased risk of cardiovascular mortality and heart failure rehospitalization in the short-term follow-up.

## Background

As a fatal and malignant disease, heart failure (HF) is becoming an epidemic that poses significant clinical and economic challenges [1]. Cardiac fibrosis, characterized by excessive intracardiac fibroblast accumulation and deposition of extracellular matrix proteins, is a fundamental process leading to myocardial structural remodeling in the failing heart, accelerating the progression to HF [2]. Inflammation, provoked by biomechanical forces or an increasing collagen deposition in the myocardial interstitium [3], stimulates the activity of cardiac fibroblasts and is considered the fundamental driving force of cardiac fibrosis [4].

Soluble suppression of tumorigenicity 2 (sST2), a powerful independent predictor of mortality in patients with HF [5], is reported to possess two different functions: anti-inflammatory activity [6] and pro-fibrotic activity promoting pathological cardiac remodeling [4, 7] by acting as a nonfunctional decoy IL‐33 receptor. The latter mechanism renders IL-33 unavailable to bind membrane-bound ST2 receptors (ST2L), thus limiting IL‐33/ST2L signaling [8]. However, in the Framingham Heart Study, sST2 was not associated with echocardiographic findings of remodeling [9] and there was no correlation between sST2 levels and cardiac fibrosis, as detected by late gadolinium enhancement on cardiac magnetic resonance imaging (CMRI), in myocarditis patients [10]. Furthermore, the sST2 level in the circulation was reported to not correlate with cardiac fibrosis in patients with HF [11].

We hypothesized that the primary cause of increased sST2 levels in patients with HF is the anti-inflammatory response induced by biomechanical forces and that its pro-fibrotic effect is just a by-product of this response. This study was designed to test the hypothesis that the ratio of sST2/left ventricular mass index (LVMI), which is a novel parameter of the prefibrotic inflammatory phase of HF that adjusts for the cardiac-remodeling effect of circulating sST2 [12, 13], is associated with prognosis in HF with reduced ejection fraction (HFrEF). LVMI was assessed using the standard CMRI technique.

## Methods

### Study population

We conducted a prospective cohort study at the Department of Cardiology, Zhongshan Hospital of Fudan University, Shanghai City, China, from September 1, 2015, to December 31, 2016. Patients with HFrEF were prospectively evaluated for inclusion in this study. HFrEF was diagnosed according to the current consensus statements of the American Heart Association [1] and the 2018 Chinese guidelines for the diagnosis and treatment of heart failure [14]. The inclusion criteria were as follows: (1) symptoms or signs of HF, (2) N-terminal prohormone of brain natriuretic peptide (NT-proBNP) > 125 ng/L; (3) left ventricular ejection fraction (LVEF) < 40%; and (4) New York Heart Association (NYHA) functional class ≥ II. The exclusion criteria were: (1) congenital heart disease, (2) acute coronary syndrome in the last 30 days, (3) pericardial disease, (4) pacemaker or other conditions precluding patients from CMRI, (5) severe anemia (hemoglobin < 7 g/dL), (6) chronic obstructive pulmonary disease, GOLD stage 3 or 4, and (7) estimated glomerular filtration rate < 30 mL/min/1.73 m^2^. The study protocol conformed to the Declaration of Helsinki, and its subsequent amendments and was approved by the local ethics committee of Zhongshan Hospital, Fudan University. All participants provided written informed consent.

### Collection of clinical, echocardiographic, CMRI, and biochemical variables

Covariates in the present study included general information, demographics, variables that could affect the ratio of sST2/LVMI or cardiac mortality, and HF hospitalization, based on our clinical experiences and previous reports.

Demographic data and clinical variables, including age, sex, body mass index, diastolic blood pressure, systolic blood pressure, heart rate, NYHA functional class, medical history, and cardiovascular risk factors (smoking, hypertension, and diabetes mellitus), were collected. Fasting venous blood was collected within 12 h after admission. After centrifugation at 3000 rpm for 15 min, the plasma was extracted and stored at 80 °C. Biochemical variables, including hematocrit, hemoglobin, white blood cells, NT-proBNP, sodium, creatinine, blood urea nitrogen, serum uric acid, albumin, total bilirubin, total cholesterol, high-density lipoprotein cholesterol, and hypersensitive C-reactive protein were measured. Serum biomarkers of myocardial fibrosis (sST2, procollagen III amino terminal propeptide [PIIINP], procollagen type I carboxy-terminal propeptide [PICP]) were assayed simultaneously using the respective ELISA kits. The characteristics of the assays were as follows: sST2 ELISA (Critical Diagnostics, San Diego, CA, USA, Catalog No. BC-1065E): average intra-assay coefficient of variation (CV) of 5.1%, detection limit of 1.8 ng/mL; PIIINP ELISA (MyBioSource, San Diego, CA, USA; Catalog No. MBS703383): intra-and inter-assay CV of less than 10%, detection range 0.125–8 ng/mL; PICP ELISA (Elabscience, Wuhan, China; Catalog No. E-EL-H6030): intra- and inter-assay CV less than 10%, detection range 0.78 to 50 ng/mL. Serum levels of PIIINP were measured using the Roche Elecsys autoanalyzer (Cobas e602), with intra-assay CV of 1.2%–4.1%, inter-assay CV of 3.75%, and a detection range of 5 to 1200 ng/mL.

Echocardiography was performed according to the American Society of Echocardiography guidelines [15]. All participants underwent transthoracic echocardiography by board-certified physicians using a Philips iE33 ultrasound machine (Philips Medical Systems, Eindhoven, The Netherlands) equipped with S5–1 and X3–1 probes. Left atrial diameter, LVEF, left ventricular end-diastolic diameter, and interventricular septal thickness were analyzed.

As described in our previous work [16], all subjects underwent clinical CMRI scans performed by two dedicated CMRI technologists in a 1.5-T CMRI system (MAG-NETOM Area, Siemens Healthcare, Erlangen, Germany) with an 18-channel phased-array cardiovascular coil. CMRI data analysis was performed using the dedicated software Argus (Siemens Medical Solution, Erlangen, Germany) by an observer blinded to all clinical data. Left ventricular mass (LVM) was determined by tracing the epicardial and endocardial borders of each slice at end-diastole, summing the myocardial volume of all slices, and multiplying by myocardial density (1.05 g/mL) [17]. LVM was indexed to body surface area (LVMI). Other CMRI variables were measured using methods previously described [16].

### Follow-up and outcomes

Patients were followed up by telephone calls and ambulatory visits at 9-month intervals. The primary outcome was a combined endpoint consisting of HF rehospitalization and cardiovascular death. The follow-up time was calculated from the time of discharge to the primary outcome, or 9 months after discharge. Endpoints were assessed by all coauthors.

### Statistical analysis

Data were expressed as mean (standard deviation) for Gaussian distribution or median (min, max) for skewed distribution of continuous variables and as numbers and percentages for categorical variables. The χ^2^ test (categorical variables), one-way ANOVA test (normal distribution), or Kruskal–Wallis H test (skewed distribution) was used to detect the differences among patients with different sST2/LVMI ratios (tertiles). We used univariate and multivariate Cox proportional hazards regression models to test the link between the sST2/LVMI ratio and the primary outcome with three distinct models. Model 1 was an unadjusted model. Model 2 was a minimally adjusted model only for sociodemographic variables. Model 3 was a fully adjusted model. Because Cox proportional hazards regression model-based methods are often considered inadequate to address nonlinear relationships, nonlinearity between the sST2/LVMI ratio and the primary outcome was addressed using a Cox proportional hazards regression model with cubic spline functions and smooth curve fitting (penalized spline method). If nonlinearity was detected, we first calculated the inflection point using the recursive algorithm and then constructed a two-piecewise Cox proportional hazards regression model on both sides of the inflection point. Subgroup analyses were performed using a stratified Cox proportional hazards regression model. For each continuous variable, we first converted it to a categorical variable according to the clinical cut point or tertile and then performed an interaction test. Tests for effect modification of subgroup indicators were followed by the likelihood ratio test. Log-rank tests for Kaplan–Meier survival curves were performed to test the prognostic value of various sST2/LVMI ratios.

Data were analyzed using the statistical software packages R (http://www.R-project.org, The R Foundation) and EmpowerStats (http://www.empowerstats.com, X&Y Solutions, Inc, Boston, MA). All statistical tests were two-sided, and a *P*-value < 0.05 was considered statistically significant.

## Results

### Baseline characteristics and outcomes of patients with HFrEF

After a baseline evaluation, 45 patients were enrolled. After 9 months of follow-up, 15 patients had reached the primary endpoint (33.3%), of whom two patients had died and 13 had been rehospitalized due to worsening HF. No patient was lost to follow-up. We show the baseline characteristics of the selected participants in Table 1, according to the tertile of the sST2/LVMI ratio. The average age was 48 ± 14 years, and approximately 20% were women. Patients with the highest sST2/LVMI ratio (Q3) had significantly higher blood sST2 levels, and they were more likely to have been prescribed angiotensin converting enzyme inhibitors or angiotensin receptor blockers than other groups. Opposite patterns were observed for the myocardium post-contrast T1 time and LVMI. There were no differences in other serum biomarkers, echocardiographic characteristics, or CMRI measurements among the different sST2/LVMI ratio groups (all *P* values > 0.05).

**Table 1:** Baseline characteristics of HFrEF patients

	sST2/LVMI			
	Q1 < 0.24	Q2 0.24–0.39	Q3 ≥ 0.39	*P* value
Age, mean (SD), years	49.20 (16.72)	44.33 (14.87)	50.20 (15.05)	0.548
Body mass index, mean (SD) (kg/m^2^)	25.12 (4.41)	26.17 (4.23)	25.89 (3.58)	0.791
Heart rate, mean (SD) (bpm)	90.67 (27.12)	86.47 (20.11)	82.47 (13.74)	0.570
Systolic blood pressure, mean (SD) (mmHg)	128.73 (15.90)	117.07 (14.07)	124.60 (23.59)	0.221
Diastolic blood pressure, mean (SD) (mmHg)	81.53 (10.37)	79.53 (12.87)	82.73 (15.89)	0.800
Gender				1.000
Female (n, %)	3 (20.00%)	3 (20.00%)	3 (20.00%)	
Male (n, %)	12 (80.00%)	12 (80.00%)	12 (80.00%)	
NYHA functional class				0.153
II (n, %)	9 (60.00%)	8 (53.33%)	4 (26.67%)	
III–IV (n, %)	6 (40.00%)	7 (46.67%)	11 (73.33%)	
Laboratory characteristics				
Sodium, mean (SD) (mmol/L)	141.27 (2.40)	140.93 (2.60)	140.67 (3.85)	0.862
Hemoglobin, mean (SD) (g/L)	145.13 (18.30)	140.53 (17.73)	143.60 (17.99)	0.777
White blood cells, mean (SD) (109/L)	6.89 (2.27)	6.00 (2.18)	6.82 (1.75)	0.436
Total cholesterol, mean (SD) (μmol/L)	4.01 (0.74)	3.79 (1.18)	3.93 (1.56)	0.887
High density lipoprotein cholesterol, mean (SD) (mmol/L)	0.93 (0.22)	0.84 (0.27)	1.01 (0.34)	0.252
Albumin, mean (SD) (g/L)	38.43 (3.06)	38.33 (5.19)	39.93 (3.08)	0.466
Creatinine, mean (SD) (μmol/L)	87.40 (16.86)	95.13 (22.96)	103.00 (30.70)	0.222
Blood urea nitrogen, mean (SD) (mmol/L)	6.45 (1.72)	6.54 (2.23)	7.17 (2.67)	0.635
Serum uric acid, mean (SD) (μmol/L)	482.47 (155.16)	534.87 (241.30)	521.20 (128.77)	0.716
Total bilirubin, mean (SD) (μmol/L)	13.40 (4.86)	16.17 (7.24)	17.21 (10.70)	0.408
Hypersensitive C-reactive protein, median (Q1–Q3) (mg/L)	1.85 (0.40–64.80)	3.30 (0.00–51.50)	1.70 (0.40–37.80)	0.527
Hematocrit, mean (SD) (%)	43.90 (5.12)	43.19 (4.81)	43.52 (5.66)	0.932
NT-proBNP, median (Q1–Q3) (pg/mL)	2547.00 (798.10–10,743.00)	1182.00 (389.40–5919.00)	2172.00 (132.90–11,029.00)	0.320
Serum biomarkers of myocardial fibrosis				
PINP, median (Q1–Q3) (ng/mL)	45.20 (17.30–136.60)	39.70 (13.00–77.70)	33.20 (15.30–100.00)	0.342
PIIINP, mean (SD) (ng/mL)	7.24 (1.82)	7.18 (1.59)	7.13 (2.28)	0.989
PICP, mean (SD) (ng/mL)	293.79 (112.34)	308.21 (82.07)	310.64 (106.56)	0.886
sST2, mean (SD) (ng/mL)	21.61 (6.08)	30.62 (5.89)	50.28 (13.46)	< 0.001
Echocardiography				
LV ejection fraction, mean (SD) (%)	31.13 (5.40)	29.07 (6.91)	32.27 (6.80)	0.390
Left atrial diameter, mean (SD) (mm)	51.93 (5.38)	51.73 (9.74)	49.80 (6.46)	0.688
Left ventricular end-diastolic diameter, mean (SD) (mm)	65.93 (7.88)	71.67 (13.15)	67.80 (9.89)	0.324
Interventricular septum, mean (SD) (mm)	10.07 (2.34)	9.40 (1.68)	9.20 (2.04)	0.482
Cardiac MR				
Myocardium native T1 time, mean (SD) (ms)	1076.64 (33.76)	1083.01 (21.81)	1085.89 (35.39)	0.706
Myocardium post contrast T1 time, mean (SD) (ms)	419.19 (10.40)	416.41 (14.43)	399.64 (16.77)	< 0.001
Extracellular volume, mean (SD) (%)	28.99 (0.81)	29.53 (1.53)	30.11 (1.73)	0.108
LV EDV index, median (Q1–Q3), (mL/m^2^)	175.70 (128.80–352.10)	153.95 (101.40–218.50)	155.90 (96.40–1342.50)	0.405
LV ESV index, mean (SD), (mL/m^2^)	151.91 (50.06)	123.35 (39.76)	141.83 (63.07) 127.80	0.338
LVEF, mean (SD) (%)	20.07 (6.11)	22.27 (9.06)	22.07 (7.84)	0.694
RV EDV index, mean (SD) (mL/m^2^)	93.95 (18.60)	83.51 (21.11)	89.03 (30.65)	0.498
RV ESV index, mean (SD) (ml/m^2^)	66.72 (22.02)	56.09 (19.77)	65.78 (30.54)	0.430
RVEF, median (Q1–Q3) (%)	29.70 (8.10–55.10)	29.80 (18.30–49.80)	31.10 (4.00–56.60)	0.520
CI, median (Q1–Q3) (L/min/m^2^)	2.25 (1.70–10.80)	2.37 (1.54–4.97)	2.47 (1.36–6.36)	0.983
LVM index, mean (SD) (g/m^2^)	117.15 (26.36)	100.38 (24.34)	87.35 (26.12)	0.010
Lambda coefficient, mean (SD)	0.52 (0.06)	0.53 (0.07)	0.53 (0.04)	0.588
Medical history				
ACE-I or ARB				0.034
No (n, %)	12 (80.00%)	9 (60.00%)	5 (33.33%)	
Yes (n, %)	3 (20.00%)	6 (40.00%)	10 (66.67%)	
Diuretics other than MRA				0.448
No (n, %)	9 (60.00%)	6 (40.00%)	6 (40.00%)	
Yes (n, %)	6 (40.00%)	9 (60.00%)	9 (60.00%)	
MRA				0.310
No (n, %)	6 (40.00%)	9 (60.00%)	10 (66.67%)	
Yes (n, %)	9 (60.00%)	6 (40.00%)	5 (33.33%)	
Digoxin				0.099
No (n, %)	15 (100.00%)	11 (73.33%)	13 (86.67%)	
Yes (n, %)	0 (0.00%)	4 (26.67%)	2 (13.33%)	
Cardiovascular risk factors				
Smoking				0.516
No (n, %)	9 (60.00%)	11 (73.33%)	8 (53.33%)	
Yes (n, %)	6 (40.00%)	4 (26.67%)	7 (46.67%)	
Hypertension				0.695
No (n, %)	8 (53.33%)	10 (66.67%)	8 (53.33%)	
Yes (n, %)	7 (46.67%)	5 (33.33%)	7 (46.67%)	
Diabetes mellitus				0.146
No (n, %)	14 (93.33%)	12 (80.00%)	15 (100.00%)	
Yes (n, %)	1 (6.67%)	3 (20.00%)	0 (0.00%)	
Etiology				0.276
Cardiomyopathy (n, %)	15 (100.00%)	11 (73.33%)	13 (86.67%)	
Ischemic heart failure (n, %)	0 (0.00%)	3 (20.00%)	1 (6.67%)	
Valvular heart disease (n, %)	0 (0.00%)	1 (6.67%)	1 (6.67%)	

### Relationship between the sST2/LVMI ratio and the composite outcome

In this study, we constructed three models to analyze the independent effects of the sST2/LVMI ratio on the composite outcome using multivariate Cox regression analysis. The effect sizes (hazard ratios [HRs]) and 95% confidence intervals (CIs) are listed in Table 2. In the crude model, the sST2/LVMI ratio showed a positive correlation with the composite outcome (HR 1.24, 95% CI 1.03 to 1.51, *P* = 0.00258). In the minimally adjusted model (adjusted for sex and age), the results were similar (HR 1.25, 95% CI 1.02 to 1.53, *P* = 0.033), which means that for each additional 0.1-unit change in the sST2/LVMI ratio, the risk of readmission for HF increased by 25%.

**Table 2:** Relationship between sST2/LVMI and the composite outcome in different models

Variable	Crude model (HR, 95% CI, P)	Minimally adjusted model (HR, 95% CI, P)
sST2/LVMI (per 0.1 change)	1.24 (1.03, 1.51) 0.0258	1.25 (1.02, 1.53) 0.0330

Crude model we did not adjust other covariants

Minimally adjusted model we adjusted age, gender

### Nonlinearity of the sST2/LVMI ratio and the primary endpoint

**Fig. 1:**
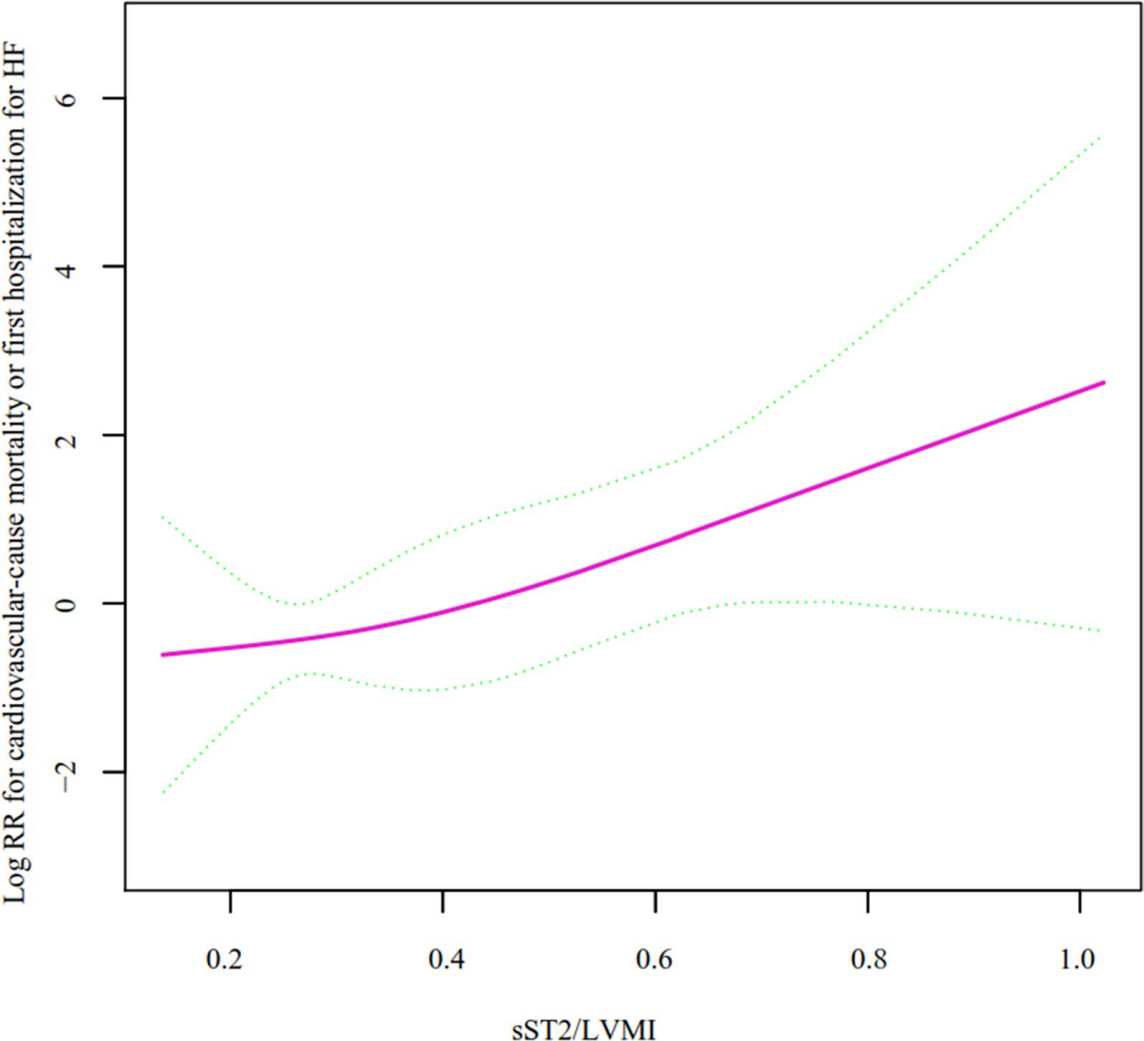
The relationship between the ratio of sST2/LVMI and the composite outcome (using the penalized spline method)

Next, we analyzed the nonlinear relationship between the sST2/LVMI ratio and the composite outcome (Fig. 1). The smooth curve and the result of the Cox proportional hazards regression model with cubic spline functions showed that the relationship between the sST2/LVMI ratio and the composite outcome was positive and linear after adjusting for sex, age, body mass index, diastolic blood pressure, systolic blood pressure, and heart rate. No nonlinear relationships were observed. The Cox proportional hazard model and the two-piecewise Cox balanced hazard model were used to fit the association based on the *P*-value from the log likelihood ratio test (Table 3).

**Table 3:** The non-linear relationship of sST2/LVMI and primary endpoint

Model 1: Fitting model by standard linear regression	
One line slope	35.06 (1.05, 1176.39) 0.0472
Model 2: Fitting model by two-piecewise linear regression	
Inflection point	0.68
< 0.68	1862.72 (0.68, 5,130,355.03) 0.0624
> 0.68	0.00 (0.00, 68,659.53) 0.4028
* P* for log likelyhood ratio test	0.199

### Results of subgroup analyses

As shown in Table 4, only a small number of interactions were observed: age, sex, systolic blood pressure, serum uric acid, and high-density lipoprotein cholesterol (all *P* values for interaction < 0.05). In the present study, stronger associations were observed in patients older than 60 years (HR 3.77 [0.93, 15.26], *P* = 0.0380), female patients (HR 4.18 [1.08, 16.16], *P* = 0.014), and for systolic blood pressure ≥ 140 mmHg (HR 3.66 [0.98, 13.65], *P* = 0.046), serum uric acid < 416 μmol/L (HR 2.43 [1.39, 4.25], *P* = 0.0052), and high-density lipoprotein cholesterol ≥ 0.9 mmol/L (HR 2.16 [1.27, 3.67], *P* = 0.0361).

**Table 4:** Effect size of sST2/LVMI on the composite outcome in prespecified and exploratory subgroups

Characteristic	No of participants	Effect size (95% CI)	*P* value	P for interaction
Age (years)				
< 60	33	1.13 (0.93, 1.38)	0.2213	0.0380
≥ 60	12	3.77 (0.93, 15.26)	0.0633	
Gender				
Female	9	4.18 (1.08, 16.16)	0.0382	0.0140
Male	36	1.17 (0.95, 1.45)	0.1446	
Systolic blood pressure (mmHg)				
< 140	35	1.20 (0.96, 1.51)	0.1139	0.0466
≥ 140	10	3.66 (0.98, 13.65)	0.0529	
Serum uric acid (μmol/L)				
< 416	14	2.43 (1.39, 4.25)	0.0018	0.0052
≥ 416	31	1.09 (0.83, 1.42)	0.5386	
High density lipoprotein cholesterol, mean (SD) (mmol/L)				
< 0.9	22	1.16 (0.86, 1.56)	0.3251	0.0361
≥ 0.9	21	2.16 (1.27, 3.67)	0.0042	

Above model adjusted for sex; age

In each case, the model is not adjusted for the stratification variable

### Predictive value of the sST2/LVMI ratio for the composite outcome in patients with HFrEF

**Fig. 2:**
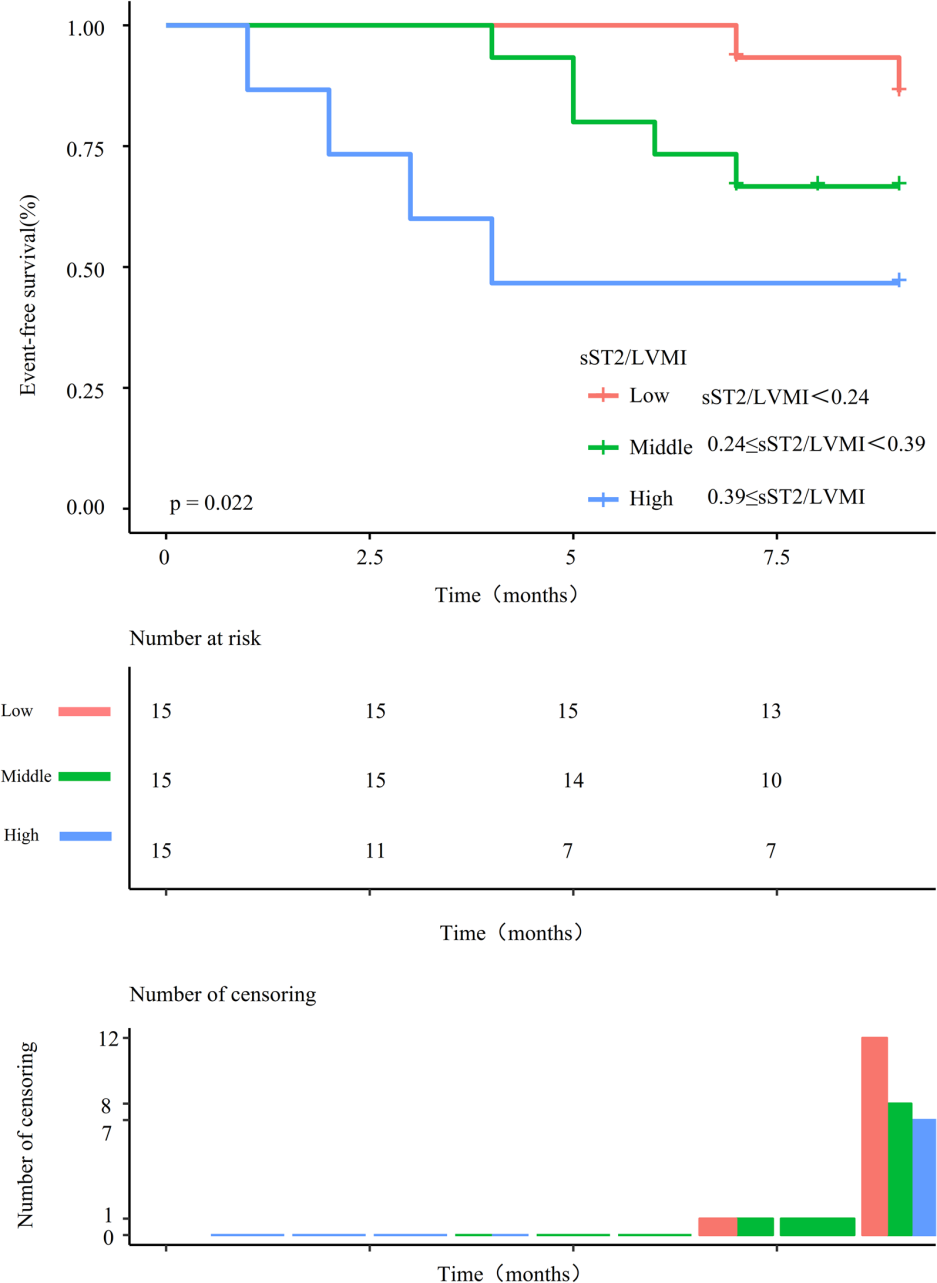
Kaplan–Meier curves showing the event-free survival in HFrEF patients according to the ratio of sST2/LVMI cut off

Kaplan–Meier curves estimated the composite outcome-free survival according to the sST2/LVMI ratio tertiles (Fig. 1). Patients with a high sST2/LVMI ratio (≥ 0.39), had shorter event-free survival than patients with an intermediate (between 0.39 and 0.24) or low (< 0.24) sST2/LVMI ratio (log-rank, *P* = 0.022). As shown in Fig. 2, there were eight, six, and two participants who reached the composite endpoint in the high, intermediate, and low groups, respectively.

## Discussion

The present study demonstrated that the sST2/LVMI ratio, which adjusts for the cardiac-remodeling effect of circulating sST2, was positively associated with the composite endpoint of cardiovascular mortality and HF readmission in Chinese patients with HFrEF. The relationship between the sST2/LVMI ratio and the primary outcome was linear. Subgroup analysis showed stronger association for patients aged between 40 and 55 years, systolic blood pressure < 115 or ≥ 129 mmHg, diastolic blood pressure < 74 mmHg, hematocrit < 44.5%, interventricular septum ≥ 8.5 mm, and right ventricular end-diastolic volume index < 74.3 or ≥ 94.3 mL/m^2^.

ST2L and sST2 are the two primary functional forms of ST2 [18]. After binding of interleukin-33 to ST2L, different intracellular signaling pathways are activated. IL-33/ST2L signaling leads to inflammatory gene transcription and the production of inflammatory cytokines/chemokines [19]. ST2L/IL-33 signaling also activates cell survival-promoting signals, resulting in several cardioprotective effects, such as inhibition of myocardial fibrosis and cardiomyocyte hypertrophy [20]. sST2, a powerful independent predictor of mortality in HF patients, acts as a decoy receptor for IL-33, rendering it unavailable to membrane-bound ST2L [21]. The biology of the ST2 system is complex, and its role in cardiovascular diseases has not been fully elucidated [22].

Cardiac fibrosis in HF patients is maladaptive and predisposes patients to cardiovascular morbidity and mortality [23]. Inflammation activated by biomechanical strain and neurohormonal factors is an important triggering and sustaining stimulus of cardiac fibrosis [24]. In terms of molecular mechanisms, sST2 is reported to possess two functions: anti-inflammatory [6] and pro-fibrotic thus promoting remodeling [4]. However, this is not supported by several clinical studies which failed to find an association between sST2 and cardiac fibrosis [9–11]. We hypothesized that the cardiac pro-fibrotic effect of elevated sST2 is a secondary effect of the inflammatory response. In the present study, we tested our hypothesis in Chinese HFrEF patients using a novel parameter, the sST2/LVMI ratio, which eliminates the cardiac-remodeling effect of circulating sST2 by adjusting for an inflammatory marker, i.e. LVMI. We measured LVMI at baseline by CMRI. We found that after adjusting for the cardiac remodeling aspect, circulating sST2 was positively associated with the composite endpoint of cardiovascular mortality and HF readmission. However, our theory needs to be explored further in future research.

Subgroup analysis can better depict the relationship between variables. As shown in Table 4, we found that sex, age, systolic blood pressure, serum uric acid, and high-density lipoprotein cholesterol were the effect modifiers of the relationship between the sST2/LVMI ratio and the composite outcome. The effect size of this relationship was magnified in female patients, older than 60 years, with systolic blood pressure ≥ 140, serum uric acid < 416 μmol/L, or high-density lipoprotein cholesterol ≥ 0.9 mmol/L. We found that all the variables mentioned above were associated with inflammation. The inflammatory response has been reported to be stronger in aging [25] and female [26] HF patients. Serum uric acid is also a marker of systemic inflammatory response in HFrEF patients [27]. The anti-inflammatory function of HDL is significantly impaired in HFrEF patients [28]. A novel finding in our study is the magnification of the relationship between the sST2/LVMI ratio and the composite outcome in patients with systolic blood pressure ≥ 140. To our knowledge, this is the first study to propose that the cardiac pro-fibrotic effect of elevated sST2 is just a secondary effect of the inflammatory response. This information may be applicable to clinical indications of ST2-related drugs in the future. Furthermore, this is the first report of an independent association between the sST2/LVMI ratio and cardiac death/HF rehospitalization in patients with HFrEF, linking this marker to important clinical outcomes. Our findings could help researchers establish diagnostic or predictive models of HF readmission or cardiovascular mortality for HFrEF patients.

We tried to address the inherent limitation of an observational study, i.e. the susceptibility to potential confounding factors, by using strict statistical adjustments, addressing nonlinearity, and performing modifying factor analysis for the different subgroups.

However, some limitations remain: (1) Our study involved Chinese HFrEF patients. and our conclusions may not be universally applicable, (2) Single-center, medium-size sample data suffer from some bias. A multicenter, large-sample study is needed to verify our findings, (3) We only investigated the correlation between baseline (admission) sST2/LVMI and prognosis, and did not address the dynamic changes of the sST2/LVMI ratio.

## Conclusions

In summary, the relationship between the baseline sST2/LVMI ratio and the composite outcome was linear in patients with HFrEF. A higher baseline sST2/LVMI ratio was associated with a higher rate of cardiovascular mortality or HF readmission during the 9-month follow-up. The sST2/LVMI ratio has an independent prognostic value in patients with HFrEF.

## Data Availability

The datasets used and/or analyzed during the current study are available from the corresponding author upon reasonable request.

## Abbreviations

sST2: Soluble suppression of tumorigenicity-2
LVMI: Left ventricular mass index
HFrEF: Heart failure with reduced ejection fraction
LV: Left ventricular
HF: Heart failure
HR: Hazard ratio
CI: Confidence interval
IL‐33: Interleukin-33
CMRI: Cardiac magnetic resonance imaging
NYHA: New York Heart Association
PINP: N-terminal propeptide of type I procollagen
PIIINP: N-terminal propeptide of type III procollagen
PICP: Type I procollagen carboxyterminal propeptide
NT-proBNP: N-terminal prohormone of brain natriuretic peptide
LVEF: Left ventricular ejection fraction
